# Pharmacological activation of focal-adhesion kinase: a promising therapeutic approach in sepsis-induced cerebral injury and cognitive dysfunction

**DOI:** 10.17179/excli2025-8668

**Published:** 2025-07-18

**Authors:** Manisha Suri, Anjana Bali

**Affiliations:** 1 Laboratory of Neuroendocrinology, Department of Pharmacology, Central University of Punjab, Ghudda, Bathinda, India

## ⁯⁯⁯

Sepsis is defined by a systemic inflammatory response to infection that can result in multiple organ dysfunction and septic shock, significantly increasing the risk of death in intensive care units (Lin et al., 2024[3]; Zhang et al., 2019[10]). Sepsis-associated encephalopathy (SAE) is one of the most common comorbidities associated with sepsis that causes substantial loss of cognitive function and functional abilities. SAE is characterized by diffuse brain functions resulting from the dysregulated systemic inflammatory response due to sepsis. This condition has become the leading cause of mortality and morbidity, with long-term neurological consequences observed in survivors of sepsis (Tang et al., 2022[7]). The onset of encephalopathy sequelae in SAE patients can manifest in a range of profound effects, including cognitive impairments, psychiatric challenges, and motor retardation. These can progress to attention deficits and, in the most severe cases, even lead to coma. Globally, instances of SAE have risen by 70 %, with 45 % of reported cases associated with cognitive deficits, and over 46 % of individuals die from SAE. As per the available reports approximately 44 % survived children from septic shock display tremendous effects on cognitive score < 25 % (Catarina et al., 2021[1]; Lamar et al., 2011[2]). The multifactorial character of SAE suggests that both systemic and brain-specific factors contribute to its aetiology. Despite that, there is no specific treatment has been introduced to address the sepsis-induced cognitive impairment. Consequently, advanced treatment is imperative to investigate the underlying cellular and molecular mechanisms associated with SAE and exalt clinical outcomes.

SAE-associated clinical manifestations in brain dysfunctions are caused by complex interactions among various factors, including bacterial endotoxin and inflammatory mediators, which lead to blood-brain barrier (BBB) dysfunction, hypoperfusion, mitochondrial damage, neuro-inflammation, and neuronal apoptosis. Systemic and neuroinflammatory responses mediated by endotoxins such as lipopolysaccharides (LPS) may be the reason behind BBB disruption, which is pivotal in the pathogenesis of sepsis-induced cerebral damage (Catarina et al., 2021[1]). Endothelial injuries have been found in SAE patients, evidenced from autopsy (Lamar et al., 2011[2]). Sepsis causes cytokine activation, which further triggers inflammation and oxidative stress. Multiple studies have reported that TNF-α, IL-1β, and IL-6 are the principal inducers of the inflammatory response, which has been linked to the development of cerebral injury. Preclinical studies demonstrate a significant increase in proinflammatory cytokines in response to sepsis, underscoring the serious impact of sepsis on the immune system. Additionally, LPS coupled with TLR4 present on cerebrovascular endothelial cells triggers NF-κB through upstream signaling pathways, including MYD88 and Rho-ROCK, which further leads to the activation of endothelial cells and compromises the BBB integrity. Literature reported that activated endothelial cells degrade the extracellular matrix and basement membrane via the disruption in adhesion molecules (ICAM-1 and VCAM-1) and matrix metalloproteinase enzymes (MMP-2 and MMP-9), which results in sudden disruption in BBB integrity in SAE (Catarina et al., 2021[1]). Collectively, these events suppress the expression of tight junction proteins including claudin-5, occludin, and ZO-1, which may contribute to SAE disease progression. Impaired BBB elevates the influx of inflammatory mediators and leukocytes, which results in activation of resting microglia. Activated glial cells initiate a counter-regulatory response by producing cytokines, NOS, and ROS, as well as triggering glutamate release, which leads to the activation of astrocytes. In addition, upregulation of glial fibrillary acidic protein (GFAP) subsequently secretes cytokines and chemokines to amplify the inflammatory cascade. This all perpetuates the vicious cycle that induces neuroinflammation and neuronal apoptosis in the hippocampus and cortex, correlates with impairment in long-term potentiation, and ultimately reduces learning and recognition capabilities. Additionally, it leads to impaired synaptic vesicle release and its stability by altering the expression of synaptic proteins (synaptophysin and CAMKII), abrupt synaptic transmission, and plasticity, ultimately causing cognitive impairment (Tang et al., 2022[7]). Notably, the changes observed during sepsis-associated encephalopathy might be attributed to an altered signal transduction pathway, that plays a critical role in cell proliferation, migration, and apoptosis, including JAK/STAT, Rho/ROCK, JNK, SRC-kinase and FAK (focal adhesion kinase).

The FAK is a cytoplasmic, multi-domain, non-receptor tyrosine kinase that is abundantly expressed in the mammalian brain, especially in the cortex and hippocampus regions, which are well known for their roles in learning and memory processes. Phosphorylated FAK acts as a key regulator in cell adhesion, migration, and cell survival, which are essential for maintaining the physiology of tissues. Its dysregulation in the brain can lead to damage to the BBB functions, alter the microglial migration, exacerbate the inflammatory responses, apoptosis, and oxidative flux, and consequently promote the disease progress. In addition, FAK recruits secondary messengers to initiate multistep signaling cascades, including PI3K/AKT, MAPK/ERK, and Rho-GTPases, to govern pathological conditions including apoptosis, inflammation, and oxidative stress (Pham et al., 2018[4]). Moreover, FAK also has the potential to increase pro-survival under hypothermic conditions by phosphorylating FAK at Y397 in SH-SY5Y neural cells (Yuan et al., 2021[9]). FAK inhibition also leads to apoptosis in Neferine-insulted SH-SY5Y cells, revealing a neuroprotective role of FAK activation (Pham et al., 2018[4]). A study by Rong et al. highlights the crucial role of the pFAK in activating the Rac1/cdc42-GTPases signaling pathway. The FAK-mediated activation of triggering receptor expressed on myeloid cells 2 (TREM2) effectively restored microglial migration impaired by Alzheimer's disease (AD) in response to amyloid-beta (Aβ) (Rong et al., 2020[5]). Tang's study found that TIMP1 and the CD63/integrin β1 complex activate FAK in mice under hypoxia and inflammation. This process attenuates tight junction integrity, enhances endothelial tightness, and improves BBB stability by inhibiting RhoA activity (Tang et al., 2020[8]). Another study indicated that FAK regulates AD-like phenotypes by inactivating ERK1/2, which inhibits cytoskeletal remodeling in neurons and reduces oxidative flux by decreasing insulin resistance (Saleh et al., 2022[6]). Additionally, several studies using in-vitro and in-vivo approaches demonstrate that pFAK has significant potential to attenuate sepsis-induced endothelial cell injury and is also embroiled in endothelial functions retrieval to impede the RhoA activation through the p190RhoGAP cascade (Zhang et al., 2019[10]). Activation of FAK also impedes the inflammatory responses in multiple organs, including cardiac injury and lung injury associated with septic conditions (Lin et al., 2024[3]; Zhang et al., 2019[10]).

Based on the evidence, it can be hypothesized that activating the FAK signaling pathway may provide a potential therapeutic strategy for managing SAE. A mechanistic overview suggests that the deletion or inhibition of FAK could exacerbate SAE. Therefore, there is a necessity to develop effective FAK agonists that can induce the autophosphorylation of its tyrosine sites, facilitating downstream signaling for enhanced pharmacological responses. These responses may significantly improve cerebral injury and restore cognitive functions by stabilizing the structure of synaptic neurons and enhancing the expression of TJ proteins. Further research is required to better understand the possible role of FAK in SAE (see Supplementary information, Figure S1).

## Declaration

### Acknowledgment

The authors are thankful to the Central University of Punjab, Bathinda, India for providing research facilities and the Department of Science and Technology Science and Engineering Research Board EEQ/2022/000997, New Delhi, for their gratefulness for providing us with financial assistance. Authors also would like to thank Servier Medical ART (https://smart.servier.com/) for providing smooth platform for figure preparation.

### Conflict of interest

The authors declare that they have no known competing financial interests or personal relationships that could have appeared to influence the work reported in this paper.

## Supplementary Material

Supplementary information
