# Supplementary material for: Pharmacological activation of focal-adhesion kinase: a promising therapeutic approach in sepsis-induced cerebral injury and cognitive dysfunction
Source: EXCLI J. 2025 Jul 18;24:851–3. doi: 10.17179/excli2025-8668 (PMC12876759; doi:10.17179/excli2025-8668)
Supplement: Supplementary information [file EXCLI-24-851-s-001.pdf]

EXCLI Journal 2025;24:851-853 – ISSN 1611-2156
Received: June 21, 2025, accepted: June 28, 2025, published: July 18, 2025

Supplementary information to:

Letter to the editor:

PHARMACOLOGICAL ACTIVATION OF FOCAL-ADHESION KINASE: A PROMISING THERAPEUTIC APPROACH IN SEPSIS-INDUCED CEREBRAL INJURY AND COGNITIVE DYSFUNCTION

Manisha Suri[1], Anjana Bali[1*]

Laboratory of Neuroendocrinology, Department of Pharmacology, Central University of Punjab, Ghudda, Bathinda, India

* **Corresponding author:** Dr. Anjana Bali, Department of Pharmacology, Central University of Punjab, Bathinda-151401, India. E-mail: anjana.bali@cup.edu.in

https://dx.doi.org/10.17179/excli2025-8668

This is an Open Access article distributed under the terms of the Creative Commons Attribution License (https://creativecommons.org/licenses/by/4.0/).

EXCLI Journal 2025;24:851-853 – ISSN 1611-2156
Received: June 21, 2025, accepted: June 28, 2025, published: July 18, 2025

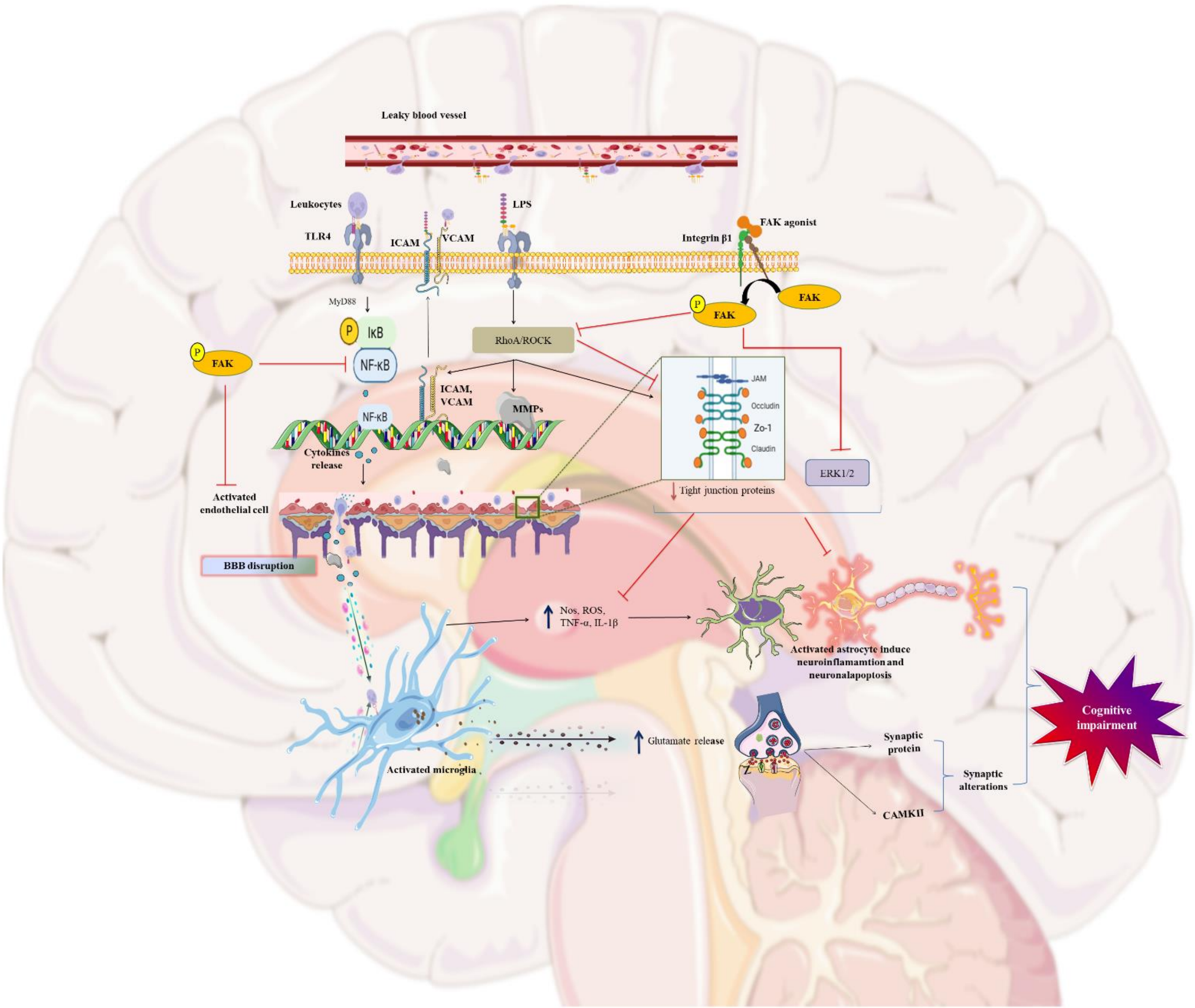

Supplementary Figure S1: Schematic representation of FAK mediated protection in sepsis associated encephalopathy and cognitive impairments. Systemic LPS undermines the integrity of the blood-brain barrier by activating cerebrovascular endothelial cells through the initiation of upstream NF-κB signalling pathways, including MyD88 and Rho-ROCK via TLR4. Moreover, ROCK activation results in elevated production of adhesion molecules like ICAM-1 and VCAM-1, as well as matrix metalloproteinase enzymes (MMP-2 and MMP-9), which annihilate tight junction proteins including claudin-5, occludin, and ZO-1. Additionally, leukocytes and inflammatory mediators incite resting microglia, initiating a counter-regulatory response characterised by the production of increased cytokines, nitric oxide synthase (NOS), and reactive oxygen species (ROS), along with the release of glutamate. This cascade activates astrocytes and modifies synaptic function by influencing the expression of pre- and post-synaptic proteins, such as synaptophysin and CAMKII, respectively. A vicious loop persists that governs neuroinflammation and neuronal death, which hinders long-term potentiation and eventually impairs learn-

EXCLI Journal 2025;24:851-853 – ISSN 1611-2156
Received: June 21, 2025, accepted: June 28, 2025, published: July 18, 2025

ing and recognition capabilities. Activation of FAK enhances cytoskeletal remodelling by obstructing neuroinflammation and apoptosis in neurones while stabilising synaptic plasticity. This effect is accompanied by the inhibition of Rho/ROCK and ERK1/2 signalling pathways, which are linked to other signalling cascades, such as NF-κB and ROS, essential for the activation of proinflammatory mediators. Furthermore, FAK enhances the production of tight junction proteins by blocking Rho/ROCK signaling.
